# Fabrication of GO/Cement Composites by Incorporation of Few-Layered GO Nanosheets and Characterization of Their Crystal/Chemical Structure and Properties

**DOI:** 10.3390/nano7120457

**Published:** 2017-12-18

**Authors:** Shenghua Lv, Haoyan Hu, Jia Zhang, Xiaoqian Luo, Ying Lei, Li Sun

**Affiliations:** 1 College of Bioresources Chemical and Materials Engineering, Shaanxi University of Science and Technology, Xi’an 710021, China; Huhaoyan0609@163.com (H.H.); Luoxiaoqian8889@163.com (X.L.); leiying0623@163.com (Y.L.); sunli01701107@163.com (L.S.); 2 College of Environment Science and Engineering, Shaanxi University of Science and Technology, Xi’an 710021, China; zhangjia_789@163.com

**Keywords:** GO nanosheets, cement composites, cement hydration crystals, mechanical properties

## Abstract

Original graphene oxide (GO) nanosheets were prepared using the Hummers method and found to easily aggregate in aqueous and cement composites. Using carboxymethyl chitosan (CCS) as a dispersant, few-layered GO nanosheets (1–2 layers) were obtained by forming CCS/GO intercalation composites. The testing results indicated that the few-layered GO nanosheets could uniformly spread, both in aqueous and cement composites. The cement composites were prepared with GO dosages of 0.03%, 0.05% and 0.07% and we found that they had a compact microstructure in the whole volume. A special feature was determined, namely that the microstructures consisted of regular-shaped crystals created by self-crosslinking. The X-ray diffraction (XRD) results indicated that there was a higher number of cement hydration crystals in GO/cement composites. Meanwhile, we also found that partially-amorphous Calcium-Silicate-Hydrate (C-S-H) gel turned into monoclinic crystals. At 28 days, the GO/cement composites reached the maximum compressive and flexural strengths at a 0.05% dosage. These strengths were 176.64 and 31.67 MPa and, compared with control samples, their increased ratios were 64.87% and 149.73%, respectively. Durability parameters, such as penetration, freeze-thaw, carbonation, drying-shrinkage value and pore structure, showed marked improvement. The results indicated that it is possible to obtain cement composites with a compact microstructure and with high performances by introducing CCS/GO intercalation composites.

## 1. Introduction

Cement composites have been used for over 2000 years, while Portland cement was discovered nearly 200 years ago and has been used ever since [1,2]. Cement composites have been widely applied to various construction engineering projects. Improving the strength and durability of these composites has been a prominent research topic in their development process [3,4]. The key goals for cement-composite improvement are higher performance and longer durability [5,6]. There are three performance classes for cement composites: normal performance, high-performance and ultra-high performance, with respective compressive strengths of less than 100 MPa, 100–150 MPa and more than 150 MPa. All three classes have a minimum durability of 200 years [7,8,9]. The present research results indicate that the strength and durability of cement composites mainly depend on their microstructure. While the factors affecting a cement composite microstructure are very complex, the main ones are cement hydration products and their aggregation methods [10,11]. Generally, cement hydration products are predominantly amorphous solids with few crystals [12,13]. Therefore, cement composites usually exhibit an incompact microstructure with a large amount of microcracks and porosity [14,15]. The structure of a material predetermines its properties; for cement composites, a compact and even microstructure benefits the improvement of compressive strength and durability [16,17]. Nowadays, the main evaluation parameters of high-performance cement composites—for example, strength and durability indexes, as well as compressive strength, flexural strength, penetration, freeze-thaw, carbonation, drying-shrinkage value and pore-structure indexes—are closely related to the microstructure of the cement composites [18,19]. However, it is difficult to prepare the cement composites so that they exhibit a compact and even microstructure: current high-performance cement composites are mainly obtained by selecting high-quality cement, suitable supplementary cementitious materials, ultra-fine or nanoscale filling materials and high-performance reinforcing materials [20,21,22,23,24,25,26]. These methods result in the high cost of high-performance cement composites. There is an urgent need to find a cheap and convenient way of preparing cement composites to obtain both high performance and an absence of structural defects.

Previous studies showed that graphene oxide (GO) nanosheets can promote the formation of regular-shaped cement hydration products and an even-compact microstructure. Both of these have a repairing effect on cracks and pores, resulting in improved strength and durability [27,28,29]. Increasingly, research on using GO nanosheets that reinforce cement composites is receiving worldwide attention [30,31]. Many researchers have investigated the effects of dosage, chemical structure and size range of GO sheets on the cement-hydration reaction, microstructure of cement materials and mechanical properties of cement composites [32,33]. Though researchers have varying opinions on the reinforcing function of GO nanosheets [34], the common consensus is that GO can, as a nano additive, have filling, interlocking and bridgings functions between cracks and pores in cement composites and that it can promote the formation of a compact microstructure in them while visibly improving mechanical strength and durability [35,36,37,38,39]. In fact, the dispersion of GO nanosheets in cement composites is the most important issue for the further development and practical application of these composites. However, research on this problem is currently limited. In our research, we found that, due to strong van der Waals interactions between GO nanosheets, the latter easily agglomerate in both aqueous solution and cement paste. This results in the uneven distribution of the GO nanosheets both in its aqueous suspension solution and in cement paste, leading to the formation of an uneven microstructure that severely influences the mechanical properties and the durability of the composite. Based on a structural analysis of GO nanosheets, we posit that the preparation of individual few-layered GO nanosheets in suspension solution is the basic premise for their uniform distribution in cement composites. In previous research on cement composites, we investigated the dispersing effects of polycarboxylate superplastisizer (PCs) [40], polyacrylic acid (PAA) [41] and grafting a polymer onto dispersed GO nanosheets [42]. The results indicated that the formation of PCs/GO or PAA/GO composites cannot uniformly distribute GO nanosheets in cement composites due to poor intercalation and dispersion capacity of PCs and PAA. The results also showed that grafting a polymer onto GO nanosheets would decrease active groups on the GO nanosheets resulting in reducing the template effects, the control capacity for cement hydration products [42].

In this paper, the GO nanosheets, which can be few-layered nanosheets and uniformly distribute in cement composites, were prepared by forming carboxymethyl chitosan (CCS)/GO intercalation composites. Following this, a series of GO/cement composites were prepared by incorporation of CCS/GO composites and investigated their microstructure, mechanical properties and durability indexes using various techniques. Our research results contribute to the preparation of high-performance cement composites through the few-layered GO nanosheet based formation of a compact and even microstructure.

## 2. Experimental Section

### 2.1. Chemicals and Materials

Carboxymethyl chitosan (CCS) is water-soluble and its average molecular mass (*M*_w_) and average molecular weight (*M*_n_) are 23,152 and 16,573, respectively. Polycarboxylate superplasticizer (PCs) was a common commercial product, supplied by Youbang New Materials Technology Co. Ltd. (Xi’an, China), with a water-reducing ratio of 33.5%. The Portland cement (P.O.42.5) was produced by Shengwei Cement Co. Ltd. (Xi’an, China). The main chemical compositions and physical parameters are shown in Table 1.

### 2.2. Preparation of GO Nanosheets Suspension

A 1000 mL round flask was placed in an ice bath at 3–5 °C and 3 g of graphite, 70 g of concentrated H_2_SO_4_ and 2 g of NaNO_3_ were added and mixed thoroughly. Then, 11 g of KMnO_4_ was gradually added to the flask over a 60 min period with stirring, kept at 3–5 °C for 1.5 h and at 35 °C for 5–6 h. After this, 120 mL of deionized water was added and kept at 80 °C for 1.5 h, following which 25 g of H_2_O_2_ was dripped into the flask over a 30 min period. The final product was purified by centrifugation, precipitation and repeated washing using deionized water until the pH of the washing water was 7.0. The graphite oxide was further treated with ultrasonic processing for 60 min. The final, original GO nanosheets suspension was obtained; the control GO content was 0.5% and the pH value was 7.0.

### 2.3. Planning and Preparation of GO/Cement Composites

The GO/cement composites consisted of cement, sand, water, PCs, CCS and GO nanosheets with the distribution shown in Table 2. The dosages of GO, CCS and PCs were solid dosage. The PCs and GO used in this study were a PCs solution and GO nanosheets suspension solution. Their water contents counted toward the total water weight. The preparation procedure consisted in first mixing water, the CCS solution and the GO suspension solution, before treating the mixture with ultrasonication for 50 min in order to prepare the CCS/GO intercalation composites. Next, the cement, sand and PCs were added to the CCS/GO intercalation composites and stirred so as to prepare the GO/cement composites. The fresh cement paste was poured into different molds to prepare test samples. After 24 h, the samples were removed from their molds and cured at 20 °C and 90% relative humidity prior to testing.

The cement composite samples included the hardened cement paste and mortar. For convenience of analysis and discussion following, using S_1_ present cement paste without GO; using S_2_, S_3_ and S_4_ present cement paste with 0.03%, 0.05% and 0.07% GO dosage, respectively; using S_5_ and S_6_ present mortar with 0.05% GO dosage and without GO, respectively.

We investigated the effects of GO nanosheets on cement composites by incorporating CCS/GO composites into the cement paste and the mortar, separately. Our initial evaluation criterion was the compressive and flexural strength after 28 days. First, we looked at the effect of the GO dosages on the strength of the hardened cement paste by doping 0.03%, 0.05% and 0.07% GO nanosheets. The compressive and flexural strength reached the maximum value at a 0.05% GO dosage. Then, we designed the mortar with this 0.05% dosage and investigated its performance.

### 2.4. Test Methods

The chemical structure of GO nanosheets was tested using a Bruker EQUINOX-55 Fourier-transform infrared spectroscopy (FTIR, Ettlingen, Germany) and a Kratos XSAM 800 XPS (Manchester, UK). The test samples were repeatedly washed to remove any other components.

The micromorphology of the GO nanosheets was measured using an SPI3800N/SPA400 AFM (Osaka, Japan). The samples were a pure single component. They were prepared by putting a drop of GO-suspension solution (0.5% GO was diluted 1000–2000 times) on a piece of monocrystalline silicon and drying it naturally. The three-dimensional shape of the GO nanosheets was obtained from the atomic force microscopy (AFM) images. The GO nanosheets’ size distribution in the suspension was obtained using a NANO-ZS90 laser particle analyzer (LPA, Worcestershire, UK). The x-ray powder diffraction (XRD) patterns of the GO and CCS/GO composites were obtained using a D/max 2200PC x-ray diffraction machine (XRD, Osaka, Japan) and the samples were prepared by freeze drying 0.5% of the original GO nanosheets suspension and CCS/GO composites.

The microstructure of the GO/cement composites was tested using a Hitachi S-4800 SEM (Tokyo, Japan). The test samples were dried and coated with gold for conductivity. The pore structure of the GO/cement composites was tested using an Autopore IV9500 automatic mercury porosimeter (Norcross, GA, USA). The samples were approximately 1 cm large. They were dried before being accurately weighed and placed in an expansion joint. They were then sealed and tested at low pressure (0–30 MPa) and then at high pressure (30–400 MPa). The crystalline structure of the composites was tested using the same XRD tester. For the S_5_ and S_6_ samples, the sand in the composites was removed for XRD testing.

The compressive strength of GO/cement composites was tested using a JES-300 concrete compressive strength tester (Wuxi, China) with an increase rate of 2.4–2.6 MPa/s. The flexural strength was determined using a DKZ-500 concrete three-point flexural strength tester (Wuxi, China) with an increase rate of 1 MPa/s. We tested five samples for each cement composite recipe. The test results were evaluated using standard deviation.

The durability parameters such as water-penetration resistance, freeze-thawing resistance, carbonation resistance and drying-shrinkage value were measured according to GB/T5082-2009.

## 3. Results and Discussion

### 3.1. Chemical Structure and Microstructure of GO Nanosheets

**Figure 1:**
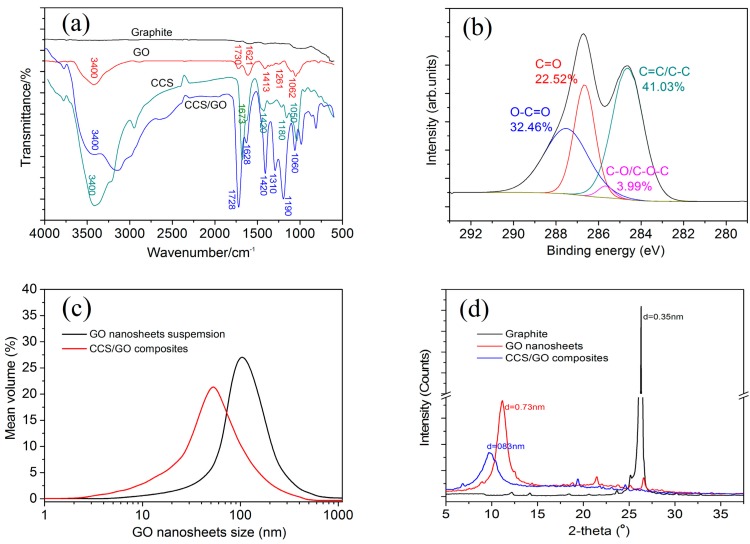
Test results of graphene oxide (GO) nanosheets. (**a**) Fourier transform infrared spectroscopy (FTIR) spectra; (**b**) X-ray photoelectron spectroscopy (XPS) spectra; (**c**) size ranges; and (**d**) X-ray diffraction (XRD) patterns of GO nanosheets.

The test results of the chemical structure and size distribution of the GO nanosheets are shown in Figure 1. Figure 1a shows the FTIR spectra of the graphite, GO, CCS and CCS/GO. The results of GO indicate that there are chemical groups of C=C (1621 cm^−1^), C–OH (3400 cm^−1^), C–O–C (1261 and 1061 cm^−1^), C=O and COOH (1730 cm^−1^). The characteristic groups of CCS are 3400 cm^−1^ (–OH and –NH_2_), 1678 cm^−1^ (–C=O) and 1420, 1180 and 1050 cm^−1^ (–O–). The results also confirmed that the CCS/GO contain these characteristic functional groups in both the CCS and GO, whose included peaks are at 3400 cm^−1^ (–OH and –NH_2_), 1728 cm^−1^ (–COOH), 1628, 1420, 1190 and 1310 cm^−1^ (–NHCO–) and 1060 cm^−1^ (–O–). FTIR spectra thereby prove that the CCS/GO samples contain both CCS and GO. Figure 1b shows the x–ray photoelectron spectroscopy (XPS) spectra of GO, suggesting that it also contains C=C/C–C, C–O/C–O–C and –C=O groups, with proportions of 41.03%, 3.99%, 22.52% and 32.46%, respectively. Figure 1c shows the size range of GO nanosheets obtained through LPA. The results indicate that the size range of GO nanosheets in CCS/GO composites is 2–380 nm, while the size range in the original GO suspension solution is 12–550 nm. The size range has clearly decreased as a result of the formation of CCS/GO intercalation composites. The main reason for the difference between two GO nanosheets is that the GO nanosheets in CCS/GO composites have been separated as individual few-layered nanosheets due to CCS penetrating into interlayers of GO nanosheets and extending the interlayer space. Figure 1d shows the XRD patterns for graphite, GO and CCS/GO. The intensity and shape of the peaks have a decreasing tendency from graphite through GO to CCS/GO. The interlayer spaces of the GO nanosheets increase from 0.35 nm of graphite to 0.73 nm of GO and 0.83 nm of CCS/GO. The results suggest that the decreased regularity of the graphite sheets is due to the oxidation and intercalation of CCS. This introduced oxygen functional groups via oxidation before inserting CCS polymer chains into the GO interlayers via intercalation, resulting in the increased interlayer spaces and the weakened interaction of the interlayers. The synergy between oxidation reaction and CCS intercalation will therefore contribute to enlarging interlayer spaces and will, to the best of its abilities, distribute the few-layered nanosheets uniformly and individually throughout the aqueous composite.

**Figure 2:**
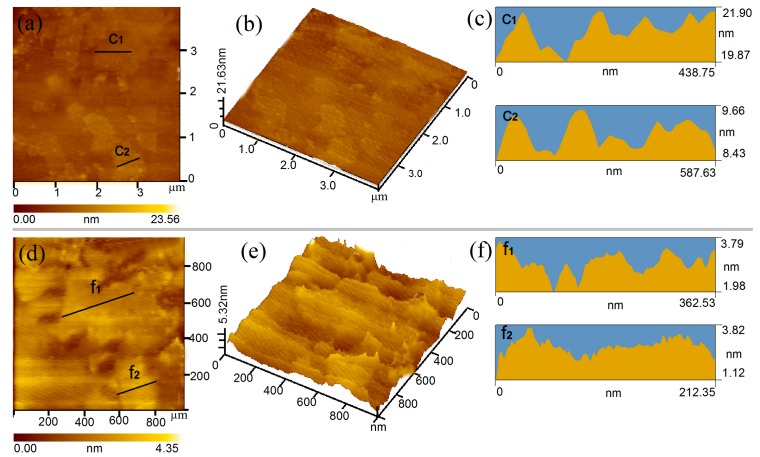
Atomic force microscopy (AFM) images of GO nanosheets: (**a**) from original GO nanosheets; (**b**) three-dimensional images; (**c**) profile images; (**d**) from carboxymethyl chitosan (CCS)/GO intercalation composites; (**e**) three-dimensional images and (**f**) profile images.

The microstructures of GO nanosheets are shown in Figure 2. Figure 2a,d exhibit the planar morphology of the original GO nanosheets and CCS/GO composites in a clear way. The size ranges of the GO nanoshets in Figure 2a,d were 200–980 and 50–450 nm, respectively. The results suggest that original GO nanosheets are larger when compared to the CCS/GO composites. The three-dimensional (3D) morphology in Figure 2b,e indicate that the original GO nanosheets have a dense and flat morphology, while the CCS/GO composites have a puffy appearance, flattening uneven surfaces. Figure 2c,f are profile views of GO and CCS/GO and their thicknesses are about 19.87, 8.43, 1.98 and 1.12 nm each. Given that their corresponding interlayer spaces are, respectively, 0.73 and 0.85 nm, their thicknesses consisted respectively of 16, 7, 2 and 1 layer of single GO nanosheets (0.35 nm). These results therefore confirm that GO nanosheets in CCS/GO composites can be few-layered (fewer than 3 layers) and uniformly and individually distributed in the aqueous composite solution. Our results suggest that GO nanosheets have an even dispersion in CCS/GO intercalation composites.

**Figure 3:**
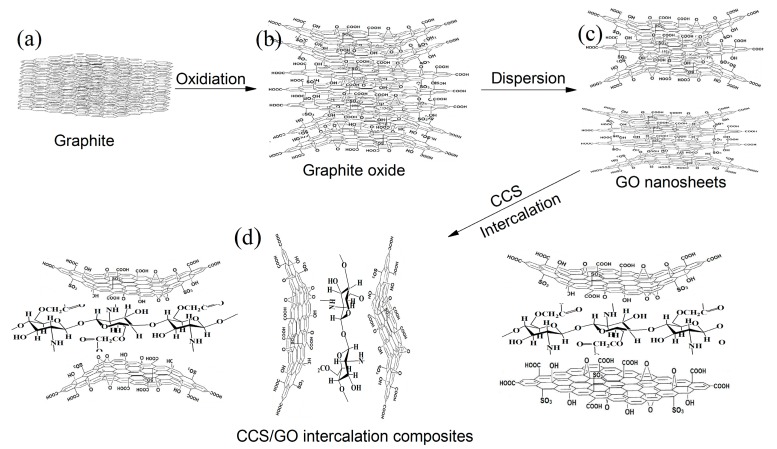
Formation process of CCS/GO intercalation composites. (**a**) Graphite; (**b**) Graphite oxide; (**c**) Graphene oxide; (**d**) few-layered graphene oxide nanosheets aggregation.

The above results indicate that GO nanosheets can disperse into 1 or 2 single layers by forming CCS/GO intercalation composites. The structure of CCS contains carboxyl groups and amine groups, which exhibit an amphoteric character. CCS has a potentially high adsorption capacity due to its positive amino groups. We show a possible working mechanism for CCS/GO intercalation in Figure 3. Figure 3a shows that graphite that is oxidized by a strong oxidizer will transfer into graphite oxide. Figure 3b shows the expansion of graphite due to chemical groups grafted on the graphite surface and the edges and the swelling first occurs at the edges. In the original GO nanosheets suspension, the GO nanosheets struggled to survive as single and individual GO nanosheets because of self-agglomeration and of the stronger interaction in the layers (Figure 3c). When CCS is added to a GO nanosheets suspension, it can penetrate the GO nanosheets and form an action between the GO and CCS because of the multiple functional groups of CCS (–NH_2_, –COOH, –OH) and the GO surface (–COOH, –OH). Moreover, CCS comprises longer chains with ring-structure units that are highly soluble. This makes it easier for them to stick to the GO nanosheets’ surface, facilitating an even dispersion in the solution, thanks to both steric hindrance and electrostatic repulsion (Figure 3d). GO nanosheets therefore exist as few-layered individual nanosheets that are uniformly distributed within the suspension.

### 3.2. Microstructure of Cement Composites

**Figure 4:**
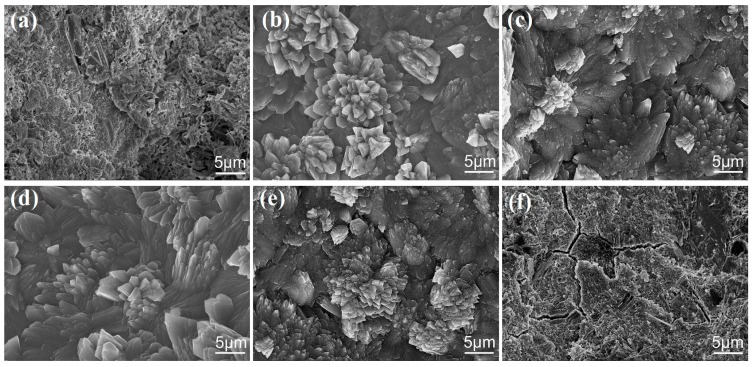
Scanning electron microscope (SEM) images of cement composites after 28 days. (**a**) S_1_; (**b**) S_2_; (**c**) S_3_; (**d**) S_4_; (**e**) S_5_; (**f**) S_6_.

After 28 days, the microstructure of the different cement composites was investigated using scanning electron microscopy (SEM). Figure 4 shows the corresponding SEM images. Figure 4a shows the microstructure of the S_1_ sample, indicating that the microstructure is an amorphous solid with many pores and microcracks. The results indicate that cement hydration products are mainly amorphous solids that form an incompactness microstructure. Figure 4b shows S_2_’s microstructure, indicating that the cement composites consist of regular-shaped crystals formed by self-interweaving and self-crosslinking. The results suggest that GO can promote the production of more regular-shaped crystals and the formation of large-scale microstructure that have an even structure. Figure 4c–e show the SEM images of S_3_, S_4_ and S_5_. One can see that these cement composites also have a similar appearance and microstructure to S_2_ but their crosslinking microstructure is more compact than that of S_2_. The results indicate that the cement composites produce a higher number of regular-shaped crystals that participate in forming the crosslinking and interweaving microstructure. This phenomenon is due to the large GO dosages in S_3_ (0.05% GO) and S_4_ (0.07% GO). Figure 4f shows S_6_’s SEM image, where one can see that the microstructure is an amorphous solid with cracks. When compared with previous research results [24,25], the above results indicate that cement composites that are composed of 0.03%, 0.05% and 0.07% of GO nanosheets obtained through CCS/GO intercalation composites have a higher amount of regular-shaped crystal products and form large-scale compact microstructures. The difference between the current preparation and previous assays is that the present experiment made use of few-layered GO nanosheets, while previous experiments used more layers GO nanosheet suspensions in cement composites. GO nanosheets in CCS/GO intercalation composites are few-layered and can be uniformly and individually distributed in cement composites. This produces more regular-shaped crystals and large-scale compact microstructures through the self-interweaving and self-crosslinking of crystals. The results suggest that the dispersion of GO nanosheets in cement composites is heavily influenced to cement crystals, particularly the macrostructure of cement composites. Improving the dispersion of GO nanosheets in cement composites would certainly be beneficial to the formation of compact structures.

**Figure 5:**
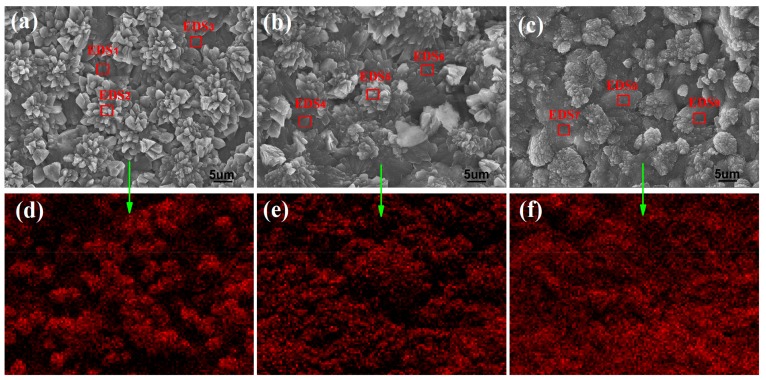
Carbon mapping in whole scanning electron microscopy (SEM) images. (**a**) S_2_; (**b**) S_3_; (**c**) S_4_; (**d**–**f**) Carbon mapping.

The above results indicate that cement composites with a compact and even microstructure can be obtained by uniformly distributing few-layered GO nanosheets in cement composites. The distribution of GO nanosheets in cement composites can be characterized by testing both the carbon mapping in a whole SEM image and the carbon content in a restricted area, using energy dispersive x-ray spectrometry (EDS). Figure 5a–c show the SEM images of S_2_, S_3_ and S_4_ samples. Figure 5d–f show the carbon mapping of the corresponding whole testing area of Figure 5a–c, respectively. The results indicate that the carbon mapping is uniformly distributed within the whole testing area. They suggest that GO nanosheets with dosages of 0.03%, 0.05% and 0.07% can uniformly and individually spread in cement composites, resulting in the formation of even and compact microstructures in whole cement composites.

The carbon content in a restricted area is shown in Table 3. The EDS testing areas are marked as red boxes in Figure 5 and the test results are shown in Table 3. The results indicate that the carbon content exhibits a gradual upward trend from S_2_ through S_3_ to S_4_. The main reason for this is that the GO dosage gradually increases from 0.03% for S_2_ through 0.05% for S_3_ to 0.07% for S_4_. The EDS test results indicate that the carbon contents in cement composites are greater than the corresponding GO dosages and the reason might be that the GO nanosheets are dispersed mainly in the crystal surface. Furthermore, the results indicate that the oxygen, silicon and calcium are also uniformly distributed in the testing areas according to their content, suggesting that the crystals have an even element composition and crystal phase structure. Finally, the carbon content at the center of the flower-like crystals is slightly higher than that of other parts such EDS_2_ and EDS_5_. The results suggest that the GO nanosheets exist mainly in initial producing crystals, located in the center position. These initial crystals may serve as a growing template for the subsequent production of further crystals. Therefore, all elements have a uniform distribution in the cement composites and this result in the production of more regular-shaped crystals and a compact microstructure.

### 3.3. Crystal Structure of Cement Hydration Crystals

The above results indicate that cement hydration products can be converted into regular-shaped crystals and that they form a compact microstructure by doping GO nanosheets. These results are a significant departure from the traditional view on cement hydration products. The main components of cement are C_3_S, C_2_S, C_3_A, C_4_AF and SCH_2_. It can react with water to produce hydration products of ettringite (AFt) [(Ca_6_Al_2_(SO_4_)_3_)(OH)_12_·26H_2_O], monosulfate (AFm) [Ca_4_Al_2_(OH)_2_·SO_4_·H_2_O], calcium hydroxide (CH) [Ca(OH)_2_], and calcium silicate hydrate (C–S–H) [3CaO·2SiO2·3H2O], gel. Generally, these hydration products can exhibit various shapes and form irregular aggregations, resulting in the formation of a microstructure with cracks and pores. The crystal structure of cement hydration products was measured using XRD.

**Figure 6:**
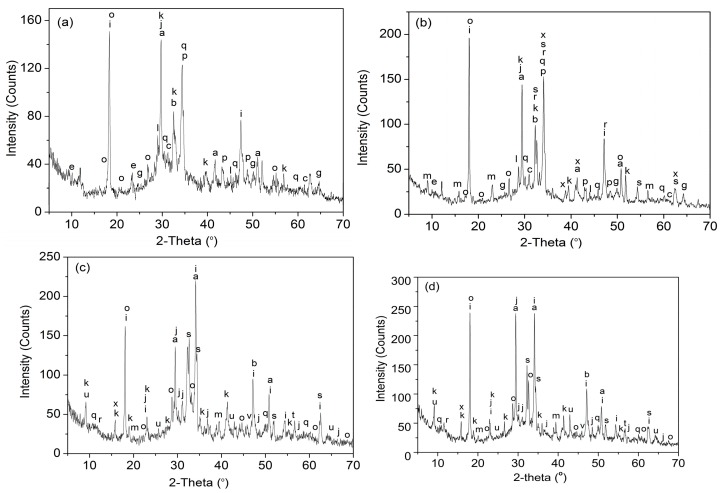
X-Ray Diffraction (XRD) patterns of cement composites at 28 days: (**a**) S_1_; (**b**) S_2_; (**c**) S_3_; (**d**) S_4_.

The XRD patterns of the cement composites are shown in Figure 6 and the analytical results are shown in Table 4. The results indicate that the cement hydration products in S_1_ (cement composites without GO nanosheets) are mainly CH, CaCO_3_, AFt, AFm, C–S–H, CaAl_2_Si_6_O_16_·6H_2_O, Ca_6_(AlSiO_4_)_12_·30H_2_O and CaHSi_2_O_7_. These products mainly exhibit crystal structure, and most products are amorphous solids and a lesser amount of regular shaped crystals. Therefore, S_1_ as a whole exhibits an amorphous solid. For the GO/cement composites of S_2_ (0.03% GO), S_3_ (0.05% GO) and S_4_ (0.07% GO), there are more cement hydration crystal products, such as CH, CaCO_3_, AFt, AFm, C–S–H, CaAl_2_Si_6_O_16_·6H_2_O, Ca_6_(AlSiO_4_)_12_·30H_2_O, CaHSi_2_O_7_, Ca_3_Si(OH)_6_(CO_3_)(SO_4_)·12H_2_O, Ca_4_Si_4_O_4_(OH)_24_·3H_2_O,Ca_5_Si_16_O_16_(OH)_2_, K_2_Ca_5_(SO_4_)_6_·H_2_O, Ca_2_Al_2_Fe_2_O_5_ and Ca_3_Si(OH)_6_(CO_3_)(SO_4_)·12H_2_O. The crystals exhibit hexagonal, cubic and tetragonal crystal structures. As they are similar to S_4_ and S_1_ respectively, the XRD patterns of S_5_ and S_6_ and their analysis results are not listed here.

Additionally, the intensity of crystal peaks in Figure 6 gradually increases with the GO dosage, from 0.03% (S_2_) through 0.05% (S_3_) to 0.07% (S_4_), which suggests that the control capacity of GO nanosheets is closely related to the GO dosage. The crystal integrity and the peak intensity show an upward trend from S_2_ through S_3_ to S_4_. We also found that the amorphous C–S–H gel can turn into monoclinic crystals in S_2_, S_3_ and S_4_. The results indicate that GO nanosheets can turn cement hydration products into regular-shaped crystals and form compact microstructures.

### 3.4. Formation Mechanism of Cement Hydration Crystals and Ordered Microstructure

**Figure 7:**
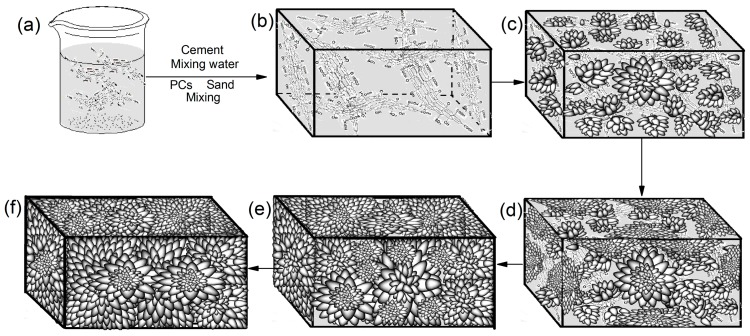
Forming mechanism of regular-shaped cement hydration crystals and compact microstructure. (**a**) CCS/GO intercalation composites; (**b**) Few-layered GO nanosheets uniformly distributed in cement paste; (**c**) The nascent crystals growing on the GO nanosheets’ template; (**d,e**) crystals growing and began forming crosslinking structure; (**f**) Final compact and even microstructure.

The above results indicate that GO nanosheets can order cement components into regular-shaped crystals and form compact microstructures during the cement hydration process. The formation mechanism mainly comprises template effects and self-assembly effects, which are shown in Figure 7. Figure 7a,b shows that GO nanosheets that exist as individual few-layered nanosheets are uniformly distributed in the cement paste by adding CCS/GO intercalation composites. Figure 7c indicates that the nascent crystals are growing on the GO nanosheets’ surfaces used as the template effects. Figure 7d,e indicates that the initial crystals grow in GO nanosheets in order to form regular-shaped crystals and then continue growing to form a compact and ordered microstructure by self-assembling and self-crosslinking, as shown in Figure 7f.

### 3.5. Mechanical Properties and Durability Parameters of GO/Cement Composites

The compressive and flexural strengths of the GO/cement composites are shown in Table 5. The results indicate that the GO/cement composites have a higher compressive and flexural strength than that of the control samples. At 28 days, the compressive strength of GO/cement composites such as S_2_, S_3_, S_4_ and S_5_ is 151.62, 175.64, 166.23 and 155.46 MPa, respectively. When compared to the control samples, the increase ratios are 42.08%, 64.87%, 56.04% and 43.11%, respectively. The compressive strengths of all the GO/cement composites, at 28 days, reach the level of ultra-high-performance cement composites. Furthermore, the corresponding flexural strengths also clearly increase when compared to the control sample. At 28 days, the flexural strengths of S_2_, S_3_, S_4_ and S_5_ is 22.83, 31.67, 29.38 and 28.65 MPa, respectively. Compared with control samples, their increase ratios are 80.05%, 149.76%, 131.71% and 154.41%, respectively. The results suggest that the flexural strengths significantly increased when compared to the compressive strengths. S_2_, S_3_ and S_4_ are hardened cement paste and S_5_ is mortar and the results indicate that, at a 0.05% GO dosage, hardened cement paste has a higher compressive and flexural strength than mortar. From the upward trend of the strengths found between three and seven days and after 28 days, we find that the GO/cement composites have little strength at 3 days and exhibit an increase in strength after seven days and after 28 days. This may be caused by the fact that the hydration crystals are produced on day 1 and begin growing on day 3. Growing further, they may form a perfect crosslinking structure on days 7 and 28. The final, perfect structure would be close to completion on day 7 and fully complete on day 28. At 60 days, the strength is slightly higher than at 28 days, suggesting that the formation of a perfectly compact microstructure in GO/cement composites is a relatively long-lasting process.

The durability of cement composites depends mainly on microstructural properties such as compactness and stability. These are usually evaluated through penetration resistance, freeze-thaw resistance, carbonation resistance, drying shrinkage and pore structure. These parameters are usually used, therefore, to evaluate the durability of cement composites. Table 6 shows the durability parameters of GO/cement composites. The results compare parameter values such as seepage height, freeze-thaw mass loss, the retention rate of a relatively dynamic elasticity modulus and carbonation depth, to those of the control samples. They suggest that the durability of GO/cement composites are remarkable improved.

**Figure 8:**
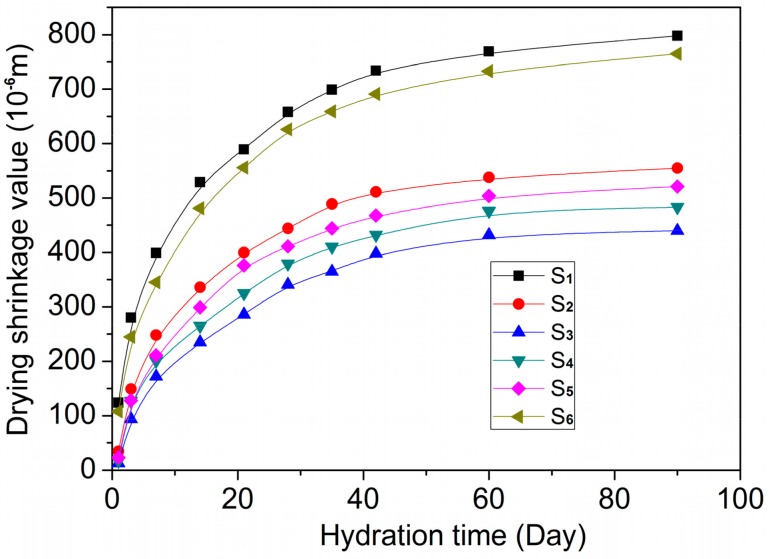
The variation of drying shrinkage value of GO/cement composites with hydration time.

The drying-shrinkage results of GO/cement composites are shown in Figure 8. The results indicate that the GO/cement composites of S_2_, S_3_, S_4_ and S_5_ have smaller drying-shrinking values when compared with the control samples of S_1_ and S_6_. When comparing S_2_, S_3_, S_4_ and S_5_, the drying-shrinkage value of S_3_ is the lowest; S_3_’s GO dosage is 0.05%, indicating that 0.05% is the optimal dosage and that the hydration products and their crosslinking structure are also the most compact and even. The results suggest that, when dosed at 0.05%, GO nanosheets have obvious inhibitory effects on the drying shrinkage of cement composites by forming compact and even microstructures. The reason for this is that GO nanosheets can control the cement hydration products, forming stable hydration crystals and a regular microstructure through the self-assembling and self-crosslinking of crystals.

The pore structure of GO/cement composites is presented in Table 7. The results indicate that the incorporation of GO nanosheets into the cement composites has an important effect on the pore structure. All GO/cement composites from S_2_ to S_5_ have a smaller total pore area, median pore diameters, average diameters and porosity when compared with the S_1_ and S_6_ control samples. The median pore diameter and average diameter of the GO/cement composites are very close and have clearly decreased when compared with the control samples. The above results indicate that it is when the GO dosage is at 0.05% that the GO/cement composites exhibit a compact microstructure, as well as the smallest average pore diameters, total pore areas and porosity. The small pores in cement composites are capillary pores and mainly attributed to free water in cement gel products [43]. GO nanosheets can transform cement hydration products into regular-shaped crystals and can form a large-scale compact microstructure through crystal growth and self-crosslinking. The crystal growth phase requires a certain porosity in order to provide space for growth, during which it will decrease the porosity. A smaller porosity is beneficial to the improvement of mechanical strengths and durability [44].

## 4. Conclusions

(1). Original GO nanosheets, prepared by Hummers’ method, are found to easily restack and aggregate in both aqueous and cement composites, a result of strong layer interactions. This causes the uneven distribution of the cement composites and a limiting of the reinforcing effects. By using CCS as a dispersant, GO nanosheets were prepared by forming CCS/GO intercalation composites; these nanosheets can exist in a few-layered form and they can uniformly spread in both aqueous and cement composites. The test results indicate that GO nanosheets can exist in aqueous composites as individual 1 to 2 layered nanosheets with a size range of 2–380 nm, while the original GO nanosheets can only exist as 7 to 16 layered nanosheets with a size range of 12–550 nm. The results therefore indicate that CCS has a strong intercalation and dispersing capacity for GO nanosheets.

(2). The cement composites that incorporated CCS/GO with a GO dosage of 0.03%, 0.05% and 0.07%, were prepared and all were found to have a compact and even microstructure. The outstanding feature is that the microstructures consist of regular-shaped crystals via self-crosslinking and self-interweaving. The EDS test results indicate that GO nanosheets can uniformly spread in the cement composites. The XRD results indicate that there are more cement hydration crystals in the GO/cement composites than in the control samples. We also found that the amorphous C–S–H gel can transform into monoclinic crystals. The results indicate that GO nanosheets are more control-effective with regards to the shaping and aggregation of cement hydration products.

(3). At 28 days, the compressive strengths of the GO/cement composites at GO dosages of 0.03%, 0.05% and 0.07% can reach 150 MPa. The maximum compressive and flexural strengths are 176.64 and 31.6 MPa at a 0.05% GO dosage, respectively. The increase ratios, when compared with the control samples, are 64.87% and 149.73%, respectively.

(4). The durability parameters such as penetration, freeze-thaw, carbonation, drying-shrinkage value and pore structure obviously improved. The results indicate that it is possible to obtain cement composites with a compact microstructure and with high performances by introducing CCS/GO intercalation composites.

## Figures and Tables

**Table 1:** Chemical and mineral compositions of the Portland cement P.O.42.5.

Chemical Components	Content (%)	Mineral Compositions	Content (%)	
Calcium oxide (CaO)	63.15	Tricalcium silicate (C_3_S, 3CaO·SiO_2_)	50.65
Silicon dioxide (SiO_2_)	21.21	Dicalcium silicate (C_2_S, 2CaO·SiO_2_)	20.32
luminum oxide (Al_2_O_3_)	6.35	Dicalcium aluminate (C_3_A, 3CaO·Al_2_O_3_)	15.63
Ferric oxide (Fe_2_O_3_)	3.34	Tetracalciumaluminoferrite (C_4_AF, 4CaO Al_2_O_3_·Fe_2_O_3_)	8.35
Alkalis (Na_2_O equivalent)	1.45	Gypsum (SCH_2_, CaSO_4_·2H_2_O)	4.27
Magnesium oxide (MgO)	2.75	f-CaO	0.78
Potassium oxide (K_2_O)	1.22			
Sulfur trioxide (SO_3_)	0.53			

**Table 2:** Components and ratios of cement composites.

Samples	Component and Composition (Weight)						Density (g/cm^3^)	Strength (MPa)	
	Cement	Sand	Water	PCs	CCS	GO		Compressive	Flexural
S_1_	100	0	20	0.6	1.0	0	2.26	116.53	10.68
S_2_	100	0	20	0.6	1.0	0.03	2.28	149.36	22.82
S_3_	100	0	20	0.6	1.0	0.05	2.29	175.64	31.67
S_4_	100	0	20	0.6	1.0	0.07	2.31	166.23	26.38
S_5_	100	80	20	0.6	1.0	0.05	2.35	155.46	29.65
S_6_	100	80	20	0.6	1.0	0	2.33	128.63	21.26

**Table 3:** Elemental composition of cement matrix doped with graphene oxide (GO).

Samples	Elemental Content (wt %)									
	C	O	Si	Ca	Al	Mg	Na	K	Fe	S
Cement	1.34	34.83	9.63	44.31	3.52	1.68	1.13	0.95	2.38	0.23
DES_1_	4.28	39.56	2.26	46.08	2.94	1.69	1.02	0.94	0.97	0.26
EDS_2_	5.63	38.54	2.51	44.91	3.41	1.65	1.15	0.65	1.32	0.23
EDS_3_	4.56	37.56	2.89	45.62	3.52	1.68	1.24	1.34	1.32	0.27
EDS_4_	6.45	41.32	2.81	42.45	2.61	1.56	1.38	0.81	0.44	0.17
EDS_5_	5.52	42.39	3.21	43.42	1.42	1.62	1.25	0.64	0.34	0.19
EDS_6_	6.65	40.49	3.21	42.39	2.56	1.78	1.12	0.78	0.81	0.21
DES_7_	9.85	40.42	2.91	40.71	1.95	1.62	1.13	0.35	0.85	0.21
EDS_8_	10.23	40.56	2.43	40.15	2.86	1.45	1.15	0.27	0.63	0.27
EDS_9_	9.94	40.55	2.35	40.81	1.98	1.68	1.35	0.35	0.76	0.23

**Table 4:** Crystal phases of cement composites.

Hydration Products	Crystal System	Cement Composites *			
		S_1_	S_2_	S_3_	S_4_
C_3_S, Ca_2_SiO_5_	Monoclinic	+	+	+	+
C_2_S, Ca_2_SiO_4_	Monoclinic	+	+	+	+
C_3_A, Ca_3_Al_2_O_6_	Orthorhombic	+	+		
C_4_AF,Ca_4_Al_2_Fe_2_O_10_	Tetrahedral	+			
CaSO_4_·2H_2_O	Monoclinic	+			
Al_2_O_3_	Hexagonal				
SiO_2_	Tetragonal	+	+	+	+
CaO	Cubic				
Ca(OH)_2_	Hexagonal	+	+	+	+
CaCO_3_	Hexagonal	+	+	+	+
AFt,Ca_6_Al_2_(SO_4_)_3_(OH)_12_·26H_2_O	Hexagonal	+	+	+	+
AFm,Ca_4_Al_2_O_6_(SO_4_)·14H_2_O)	Hexagonal	+	+	+	+
C–S–H,Ca_3_Si_2_O_7_·xH_2_O)	Amorphous	+	+	+	+
C–S–H(Ca_3_Si_2_O_7_·xH_2_O)	Monoclinic		+	+	+
CaAl_2_Si_6_O_16_·6H_2_O	Tetragonal	+	+	+	+
Ca_2_H_2_Si_2_O_7_	Orthorhombic	+		+	+
Ca_6_(AlSiO_4_)_12_·30H_2_O	Cubic	+	+	+	+
Ca_4_Si_4_O_4_(OH)_24_·3H_2_O	Monoclinic		+	+	+
Ca_3_Si(OH)_6_(CO_3_)(SO_4_) 12H_2_O	Hexagonal		+	+	+
K_2_Ca_5_(SO_4_)_6_·H_2_O	Monoclinic			+	+
CaFe_5_AlO_10_	Tetragonal		+	+	+
Ca_2_Al_2_Fe_2_O_8_	Orthorhombic			+	+

* +: The crystal phases exist in cement composites.

**Table 5:** The compressive and flexural strengths of GO/cement composites.

Samples	Compressive Strength (MPa)				Flexural Strength (MPa)			
	3 Days	7 Days	28 Days	60 Days	3 Days	7 Days	28 Days	60 Days
S_1_	40.67	75.25	106.53	117.73	3.42	8.52	12.68	13.54
S_2_	32.65	91.56	151.36	154.62	7.46	13.54	22.83	23.47
S_3_	35.41	95.75	175.64	177.36	7.85	17.28	31.67	32.46
S_4_	36.23	98.23	166.23	168.34	7.31	16.62	29.38	29.43
S_5_	31.63	87.43	155.46	158.42	6.87	14.32	28.65	27.36
S_6_	22.15	91.56	108.63	129.63	5.38	9.98	11.26	12.42

**Table 6:** Durability parameters of GO/cement composites at 28 days.

Samples	Penetration Resistance		Freeze–Thaw Cycles * (×100)			Carbonation Depth (mm)	
	Osmotic Pressure (MPa)	Seepage Height (mm)	*m*_0_ (g)	*m*_loss_ (g)	*P* (%)	7 Days	28 Days
S_1_	3.5	15.4	9837	0.55	71.52	3.73	4.94
S_2_	3.5	4.7	9833	0	89.5	2.73	3.23
S_3_	3.5	3.6	9845	0	96.53	0.84	1.84
S_4_	3.5	3.7	9836	0	98.76	0.65	1.35
S_5_	3.5	4.1	9841	0	97.65	0.52	1.62
S_6_	3.5	11.3	9851	0.45	73.34	3.53	4.34

* m_0_: the weight of samples before freeze–thaw experiments. *m*_loss_: the weight of samples after 100 freeze-thaw cycles. *P*: the retention rate of a relatively dynamic elasticity modulus of the test samples after 100 freeze-thaw cycles.

**Table 7:** Pore structure of GO/cement composites at 28 days.

Samples	Pore Structure of Cement Composites				
	Total Pore Area (m^2^/g)	Median Porediameter (nm)	Average Pore Diameter (nm)	Apparent Density (g/cm^3^)	Porosity (%)
S1	24.86	39.42	55.13	2.21	23.74
S2	16.59	22.34	21.94	2.31	17.36
S3	13.68	15.25	14.67	2.35	11.25
S4	12.32	14.32	13.45	2.33	10.25
S5	15.14	17.67	19.32	2.35	15.43
S6	27.43	45.72	45.65	2.34	21.62
